# Characteristic Eye Movements in Ataxia-Telangiectasia-Like Disorder: An Explanatory Hypothesis

**DOI:** 10.3389/fneur.2017.00596

**Published:** 2017-11-09

**Authors:** Pamela Federighi, Stefano Ramat, Francesca Rosini, Elena Pretegiani, Antonio Federico, Alessandra Rufa

**Affiliations:** ^1^Eye Tracking and Visual Application Lab (EVA Lab), Department of Medicine, Surgery and Neuroscience, University of Siena, Siena, Italy; ^2^Department of Electrical, Computer and Biomedical Engineering, University of Pavia, Pavia, Italy; ^3^Laboratory of Sensorimotor Research, National Eye Institute, National Institutes of Health, Bethesda, MD, United States; ^4^UOC Neurology and Neurometabolic Diseases, Department of Medicine, Surgery and Neuroscience, University of Siena, Siena, Italy

**Keywords:** ataxia-telangiectasia-like disorder, saccade hypermetria, granule cells, parallel fibers, Purkinje cells

## Abstract

**Objective:**

To investigate cerebellar dysfunctions and quantitatively characterize specific oculomotor changes in ataxia-telangiectasia-like disorder (ATLD), a rare autosomal recessive disease caused by mutations in the *MRE11* gene. Additionally, to further elucidate the pathophysiology of cerebellar damage in the ataxia-telangiectasia (AT) spectrum disorders.

**Methods:**

Saccade dynamics, metrics, and visual fixation deficits were investigated in two Italian adult siblings with genetically confirmed ATLD. Visually guided saccades were compared with those of 40 healthy subjects. Steady fixation was tested in primary and eccentric positions. Quantitative characterization of saccade parameters, saccadic intrusions (SI), and nystagmus was performed.

**Results:**

Patients showed abnormally hypermetric and fast horizontal saccades to the left and greater inaccuracy than healthy subjects in all saccadic eye movements. Eye movement abnormalities included slow eye movements that preceded the initial saccade. Horizontal and vertical spontaneous jerk nystagmus, gaze-evoked, and rebound nystagmus were evident. Fixation was interrupted by large square-wave jerk SI and macrosaccadic oscillations.

**Conclusion:**

Slow eye movements accompanying saccades, SI, and cerebellar nystagmus are frequently seen in AT patients, additionally our ATLD patients showed the presence of fast and hypermetric saccades suggesting damage of granule cell-parallel fiber-Purkinje cell synapses of the cerebellar vermis. A dual pathogenetic mechanism involving neurodevelopmental and neurodegenerative changes is hypothesized to explain the peculiar phenotype of this disease.

## Introduction

Autosomal recessive cerebellar ataxias with DNA-double strand break repair deficits are a group of severe neurodegenerative and systemic diseases featuring early-onset ataxia and radiosensitivity including ataxia-telangiectasia (AT), the most common disorder of this group, and ataxia-telangiectasia-like disorder (ATLD) (1). ATLD is a very rare autosomal recessive disease due to mutations in the *MRE11* gene (2). This gene encodes a protein (Mre11) with nuclease and DNA-binding activity; together with Rad50 and Nbs1, it forms the MRN complex which is a target of ATM kinase and is involved in the signaling network of cellular response to DNA damage (3, 4). To date, reports document only 23 cases of ATLD belonging to two families from the United Kingdom (one native from Pakistan), one family from Italy, three families from Saudi Arabia, three families from Japan, and one family from Pakistan (2, 5–11). The clinical features of the majority of patients with ATLD resemble those of patients with AT including progressive cerebellar ataxia, oculomotor apraxia, and cellular hypersensitivity to ionizing radiations, with a generally mild presentation and slow progression (12). Like in AT, facial dyskinesia, choreoathetosis, and dystonia may also be present; whereas telangiectasia, immunodeficiency, and increased α-fetoprotein have not been reported (8, 13). Clinical descriptions of oculomotor changes in both AT and ATLD patients show inability to initiate voluntary saccades, saccade hypometria, delayed convergence and impaired smooth pursuit, vestibulo-ocular reflex (VOR), and optokinetic nystagmus (7, 10); fixation abnormalities such as saccadic intrusions (SI), drifts, spontaneous, gaze-evoked, and down-beat nystagmus (7).

Ataxia-telangiectasia-like disorder cases with a more severe phenotype have been observed: four subjects from two Saudi Arabian families showed microcephaly, as well as two unrelated patients from Japan who presented also a bird-headed facial appearance, mental retardation, no cerebellar ataxia or oculomotor apraxia (6, 11), and two Japanese siblings with minor dysmorphisms, cognitive delay, and lung adenocarcinoma (8). Overall these features recall Nijmegen breakage syndrome (NBS), due to mutations in Nbs1 of the MRN complex, which is characterized by microcephaly, growth retardation, immune dysfunction, and radiosensitivity with predisposition to cancer but no ataxia (3, 4). This suggests that some *MRE11* mutations could have a pivotal role during development giving rise to a wider clinical spectrum than that related solely to neurodegeneration.

The neural substrate of the network controlling eye movements is relatively well known. Therefore, the study of eye movement abnormalities, particularly in rare diseases with known genetic pathology, represents an ideal tool to investigate and model the function of discrete circuits of this network (14). The quantitative analysis of eye movement defects has not been reported in ATLD patients. Therefore, this study was principally designed to quantitatively characterize specific oculomotor changes in ATLD patients that may help to define diagnosis and contribute to better characterize cerebellar involvement in the control of eye movements. An additional purpose was to further elucidate the pathophysiology of cerebellar damage in ATLD. The main result of the study suggests that ATLD may damage granule cells (GCs) and their parallel fibers (PFs) in the cerebellar vermis. Finally, we propose a hypothetical scheme in which both neurodevelopmental and neurodegenerative components of cerebellar damage may account for the pathophysiology of the oculomotor changes observed in ATLD.

## Subjects

Two affected siblings, respectively, 45 (male, Patient 1) and 44 years old (female, Patient 2) were studied. Both wild-type for *ATM* and *NBS1*, but compound heterozygotes for *MRE11* gene mutations [1422C→A, T481K; 1714C→T, R571X]. The 1422C→A allele was inherited from the mother, whereas the paternally inherited 1714C→T allele was apparently null as a result of non-sense-mediated mRNA decay (5). Complete neurological, instrumental MRI, and cognitive investigations were obtained in these patients with a long clinical and MRI follow-up. The neuro-ophthalmological examination included visual acuity for distance and near, pupils and anterior segment evaluation, ocular alignment, nystagmus, conjugate eye movements, and ophthalmoscopy. Experimental protocols were approved by the Local Ethics Committee of the Azienda Ospedaliera Universitaria Senese and procedures performed in studies were in accordance with the 1964 Helsinki declaration and its later amendments or comparable ethical standards. Written informed consent was obtained from individual participants included in the study.

## Materials and Methods

Eye movements were measured using a video-based, remote, monocular recording, two-dimensional eye tracking technique (ASL 504, sample rate: 240 Hz) (15). The experiment was designed to study horizontal visually guided saccades (10°–18°) (16) and steady fixation in primary and eccentric positions (10°–18°), in order to study SI and nystagmus (17). Due to the head and neck dystonia which induced unbearable fatigue and poor compliance during the head constraint, patients performed a series of independent experiments on different sessions (in a period lasting 1 year). During this period, the disease remained clinically stable in both patients.

We performed a quantitative analysis of saccadic parameters of the first reflexive horizontal saccade executed in response to target presentation. We considered the following parameters: saccade duration (time interval between the start and the end of the saccade); saccade latency (time delay between target onset and saccade onset); saccade amplitude (change in eye position, in degrees of visual angle, between the start and end of the saccade); peak saccade velocity (maximum eye velocity, in degrees of visual angle/s); peak acceleration and peak deceleration (maximum eye acceleration and deceleration, in degrees of visual angle/s^2^); and saccade accuracy, based on the absolute error (modulus of difference between target position and eye position at the end of the initial saccade, in degrees of visual angle). The main sequence relationships of peak velocity and duration versus amplitude were fitted using an exponential and a linear function, respectively. The saccade onset and offset times were based on a 10°/s velocity threshold. Recorded data were processed off-line using an interactive algorithm to identify each saccade, check automatic identification, and calculate saccadic parameters (18).

Saccadic intrusions were characterized by several types of inappropriate saccadic movements identified as square-wave SI and macrosaccadic oscillations (MSO). Square-wave SI were classified according to their waveform into two different types: monophasic or square-wave jerks (SWJs) and biphasic square-wave saccadic intrusions (BSWSI) (19, 20). SWJ were characterized by pairs of horizontal saccades: a first saccade was directed away from the target and a second saccade returned to it, generally after a period of time (intersaccadic interval) ranging 200–400 ms. BSWSI were characterized by three horizontal saccades: a saccade was directed away from the target, a following hypermetric saccade was directed in the opposite direction overshooting the target and, after an interval of time ranging 200–300 ms, a corrective saccade that returned back to the target. MSO were back to back hypermetric saccades, with an intersaccadic interval of about 200 ms; they oscillated around the target and spontaneously grew larger and then smaller (in amplitude). The quantitative characterization of SI included amplitude of saccades, their intersaccadic interval, and frequency of square-wave SI (17) or frequency of oscillations in MSO. Nystagmus was characterized by alternation of slow drift of the eye position (slow phase) followed by a rapid correction (quick phase). Nystagmus was described using slow phase velocity, amplitude, and frequency of fast phases. The rapid gaze shifts that intruded on visual fixation were identified automatically and their start and end were defined as the times when eye velocity exceeded or fell below 50°/s. All responses were tested by visual inspection of waveforms in eye position signals and the analysis was corrected interactively if necessary.

Forty age-matched healthy subjects (mean age: 35 years; range: 30–60) served as controls in saccade evaluation. Estimated descriptive parameters of SI were compared with the normal values previously reported (19). Differences in means of all saccadic parameters, which were calculated for each experiment of each patient, and differences of descriptive parameters of SI between patients and the healthy subjects were analyzed by the Mann–Whitney *U* test and Spearman correlation coefficients were estimated.

## Results

### Clinical Neuro-Ophthalmological and MRI Profile

**Figure 1:**
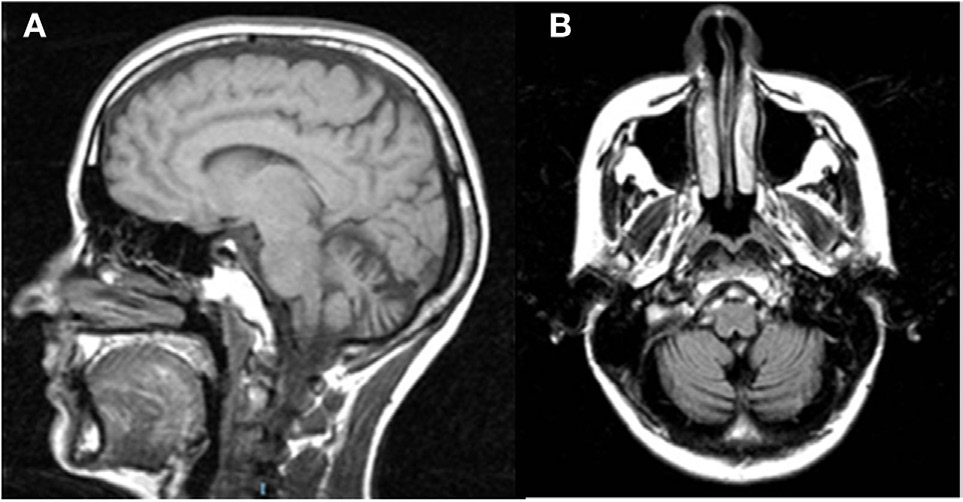
Sagittal T1w **(A)** and coronal T2w-FLAIR **(B)** magnetic resonance images of Patient 2 show vermian and hemispheric cerebellar atrophy.

Table 1 summarizes a detailed report of clinical findings; neurological, MRI (see Figure 1), and cognitive follow-up of the two siblings have been recently updated and reported (20). Neurological examination showed a common clinical pattern in our two patients with a slow disease progression until age 14, followed by a long period of clinical, neurological, cognitive, and neuroimaging stability in adulthood with only dystonia of the arms and ataxic gait slightly deteriorating. No cancer was found.

**Table 1:** Demographics, clinical, and instrumental findings of patients with ataxia-telangiectasia-like disorder.

Clinical and instrumental findings	Patient 1	Patient 2
Sex/age at examination	M/45 years old	F/44 years old
Afferent visual functions	Normal	Normal
Ocular movements examination	Fast and inaccurate saccades, disrupted pursuit, abnormal VOR	Fast and inaccurate saccades, disrupted pursuit, abnormal VOR
Eye oscillations	Spontaneous GEN and rebound ny, SI	Spontaneous GEN and rebound ny, SI
Expressionless face	++	++
Head ataxia	++	++
Dysarthria	+++	+++
Hypotonia	++	++
Dysmetria/action tremor	+++	+++
Choreo-athetotic movements	++	+
Limbs dystonia	++	–
Joint laxity	++	++
Gait ataxia (with frequent falls)	+++	+++
EMG: axonal sensory neuropathy	+	+
MRI: cerebellar atrophy	+++	+++
ICARS subscores		
Posture and gait disturbances	26	26
Kinetic functions	31	31
Speech disorders	5	5
Oculomotor disorders	6	6
Global score	68	68

Visual acuity, color vision, pupils, and ophthalmoscopy were not impaired and no conjunctival telangiectasias were evident. The evaluation of conjugate eye movements showed similar abnormalities in the two siblings (see Table 1).

### Quantitative Characteristics of Saccade Abnormalities

**Figure 2:**
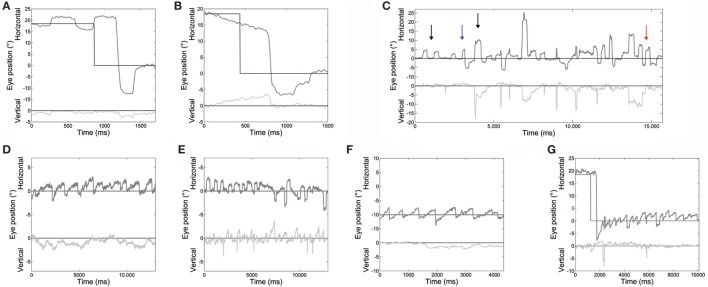
An example of different oculomotor abnormalities found in both patients. **(A)** Very large hypermetric saccade, with higher than normal speed and followed by corrective saccades to reach the target. **(B)** Slow drifts preceding a centripetal saccade. **(C)** Large square-wave saccadic intrusions: square-wave jerk, macrosquare-wave jerks (black arrows), biphasic square-wave saccadic intrusions (blue arrow); and macrosaccadic oscillations (red arrow). **(D)** Spontaneous jerk nystagmus with mainly horizontal and, sometimes, **(E)** vertical component appearing as an oblique nystagmus. **(F)** Gaze-evoked nystagmus in eccentric gaze positions of 10°, and rebound nystagmus **(G)** after the eye was returned to central position. Horizontal and vertical eye positions are plotted in different shades of gray. The black line shows horizontal and vertical target position. Positive values correspond to rightward and upward.

Main eye movement abnormalities in both patients, pointed out by the analysis of saccade and fixation parameters, are shown in Figure 2.

Saccade amplitude was greater in ATLD patients than controls for both target jumps (10°: *P* < 0.001). The mean amplitude for the initial saccades was 13.8 ± 1.3° for 10° saccades and 19.0 ± 1.5° for 18° saccades (means for controls, 10°: 10.2 ± 0.5°; 18°: 18.1 ± 0.7°). Saccadic amplitude of each patient is summarized in Table 2. The mean amplitude for the initial leftward (LW) saccades was 15.8 ± 3.9° for 10° target jumps and 22.2 ± 5.7° for 18° target jumps (means for controls, 10°: 10.3 ± 1.1°; 18°: 18.1 ± 1.7°). The mean amplitude for 10° target jumps to the right was instead 11.1 ± 3.6° and 16.5 ± 4.0° for the 18° target jumps (means for controls, 10°: 10.1 ± 1.1°; 18°: 18.1 ± 1.6°). LW and rightward (RW) saccade amplitudes were significantly different (LW 10°: *P* < 0.001; 18°: *P* < 0.001; RW 10°: *P* < 0.05; 18°: *P* < 0.001) in our patients compared with controls. An example of very large LW hypermetric saccades, typically followed by corrective saccades to reach the target, is illustrated in Figure 2A.

**Table 2:** Saccade metric parameters estimated for all saccades in each experiment.

Patients		Amplitude	Accuracy	Amplitude leftward saccades	Amplitude rightward saccades	Accuracy leftward saccades	Accuracy rightward saccades
Patient 1	10°	(14.6 ± 0.5)°	(5.9 ± 0.5)°	(17.8 ± 4.5)°	(10.7 ± 4.1)°	(8.0 ± 4.2)°	(3.2 ± 2.5)°
	18°	(19.3 ± 1.6)°	(5.8 ± 2.0)°	(25.4 ± 7.0)°	(15.4 ± 5.4)°	(8.4 ± 5.1)°	(5.1 ± 3.0)°
Patient 2	10°	(13.2 ± 1.5)°	(3.8 ± 1.4)°	(14.1 ± 2.3)°	(11.4 ± 3.2)°	(4.3 ± 2.1)°	(2.6 ± 2.5)°
	18°	(19.0 ± 1.7)°	(4.0 ± 1.9)°	(20.7 ± 4.4)°	(16.9 ± 3.0)°	(3.8 ± 3.0)°	(2.6 ± 2.1)°

*Amplitude and accuracy (absolute error value is shown) for 10° and 18° saccades for Patient 1 and Patient 2. The first two columns show the mean of means and the SEM computed over each recording session for each patient. The rightmost four columns, instead, present the mean and the SD of the indicated parameters computed by pooling all appropriate saccades from all recordings*.

Saccadic dysmetria, in which the eye overshoots or undershoots the target, was more accurately estimated using saccade accuracy. We found a higher absolute error of primary saccades (10°: *P* < 0.001; 18°: *P* < 0.001) in ATLD patients (mean, 10°: 4.7 ± 1.4°; 18°: 4.8 ± 2.0°) than in controls (mean, 10°: 0.8 ± 0.3°; 18°: 1.2 ± 0.5°). The mean absolute error was greater in Patient 1 than Patient 2. Saccadic absolute error of each patient is summarized in Table 2. The mean absolute error for the initial LW saccades was 5.9 ± 3.7° for 10° target jumps and 5.2 ± 4.3° for 18° target jumps (means for controls, 10°: 0.8 ± 0.8°; 18°: 1.2 ± 1.3°). The mean absolute error for RW saccades was 2.8 ± 2.5° for 10° target jumps and 3.5 ± 2.8° for the 18° target jumps (means for controls, 10°: 0.8 ± 0.7°; 18°: 1.2 ± 1.1°). LW and RW saccade absolute errors were significantly different (LW 10°: *P* < 0.001; 18°: *P* < 0.001; RW 10°: *P* < 0.001; 18°: *P* < 0.001) in our patients compared with controls.

Latency of saccades was prolonged, with a mean value of 296 ± 36 ms for 10° saccades and 292 ± 44 ms for 18° saccades (means for controls, 10°: 237 ± 88 ms; 18°: 241 ± 87 ms). The mean latency for the initial LW saccades was 284 ± 90 ms for 10° target jumps and 267 ± 54 ms for 18° target jumps (means for controls, 10°: 208 ± 85 ms; 18°: 220 ± 87 ms). The mean latency for 10° target jumps to the right was instead 294 ± 90 and 289 ± 97 ms for the 18° target jumps (means for controls, 10°: 215 ± 83 ms; 18°: 220 ± 76 ms). LW and RW saccade latencies were significantly different (LW 10°: *P* < 0.001; 18°: *P* < 0.001; RW 10°: *P* < 0.001; 18°: *P* < 0.001) in our patients compared with controls.

The duration of saccades to 10° target jumps was higher in patients than controls (10°: *P* < 0.001), the average values in patients were 65 ± 9 ms for 10° saccades and 72 ± 15 ms for 18° saccades (means for controls, 10°: 49 ± 5 ms; 18°: 65 ± 5 ms). The mean duration for the initial LW saccades was 69 ± 25 ms for 10° target jumps and 71 ± 24 ms for 18° target jumps (means for controls, 10°: 48 ± 9 ms; 18°: 65 ± 10 ms). The mean duration for 10° target jumps to the right was instead 58 ± 18 and 71 ± 20 ms for the 18° target jumps (means for controls, 10°: 48 ± 9 ms; 18°: 66 ± 12 ms). The duration of LW and RW saccades was significantly different in our patients compared with controls only for 10° target jumps (LW 10°: *P* < 0.001; 18°: *P* > 0.05; RW 10°: *P* < 0.001; 18°: *P* > 0.05).

Saccade peak velocity for saccades toward both eccentric targets (10°–18°) was higher in both patients than in controls, although the difference did not reach significance when we analyzed the saccade peak velocity without dealing separately with RW and LW saccades. The mean saccade peak velocity for the initial LW saccades was 467 ± 84°/s for 10° target jumps and 601 ± 116°/s for 18° target jumps (means for controls, 10°: 388 ± 65°/s; 18°: 497 ± 77°). The mean saccade peak velocity for 10° target jumps to the right was instead 356 ± 89° and 466 ± 96°/s for the 18° target jumps (means for controls, 10°: 378 ± 65°/s; 18°: 499 ± 94°). LW and RW saccade peak velocities were significantly different (LW 10°: *P* < 0.001; 18°: *P* < 0.001; RW 10°: *P* = 0.002; 18°: *P* < 0.05) in our patients with respect to controls.

**Figure 3:**
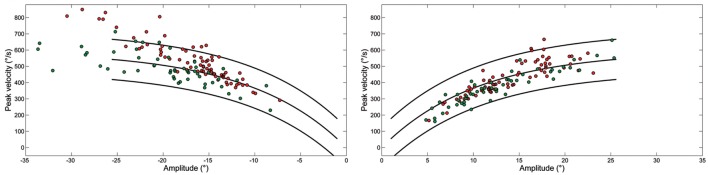
Plot of main sequence relationships between peak velocity and amplitude of saccades from Patient 1 (green) and Patient 2 (red) as data points. Curves show the main sequence relationship and 5 and 95% prediction intervals for healthy subjects. Plot of peak velocity versus amplitude of leftward (LW) and rightward saccades from each ataxia-telangiectasia-like disorder patient. Larger LW saccades made by Patient 2 often exceeded the 95% confidence interval for healthy subjects.

Figure 3 shows the peak velocity versus amplitude main sequence relationship separately considering RW and LW saccades. The main sequence relationship was fitted using the classical exponential equation for healthy subjects and their 95% confidence interval is indicated. Larger saccades of both patients, especially Patient 2, were above the confidence interval bounds for controls. The relationship between peak velocity and amplitude in patients was better fitted by a linear model. To determine the accuracy of models on the entire dataset, model prediction was evaluated using percentage root mean square error, where large values of this parameter indicated poor fit. The slope of the linear equation was greater for LW than for RW saccades in both patients. Saccade acceleration (*P* < 0.001) and deceleration (*P* < 0.001) were significantly different in patients than controls. The amplitude-normalized average values of peak acceleration and deceleration normalized to amplitude in patients were 1,682 ± 635 and 1,661 ± 868 1/s^2^, respectively, and 1,893 ± 744 and 1,866 ± 767 1/s^2^ in controls.

Slow eye movements (Figure 2B) preceded 23% of initial saccades, 17% occurring in centripetal, and 6% in centrifugal saccades. Slow movements were centripetal when the subject attempted to hold the eyes in an eccentric position and centrifugal RW when attempting to hold the eyes in a central position. Slow movements were opposite with respect to the direction of upcoming saccades in 22% of cases. The mean amplitude of these slow eye movements was 2.9 ± 0.6°. Their velocity profile was relatively linear, with a mean velocity of 5.4 ± 1.6°/s. Their velocity also decreased with the reduction of eye position eccentricity, the mean velocity versus target eccentricity was: 3.9 ± 1.5°/s at 0°; 5.5 ± 0.8°/s at 10°; and 6.9 ± 1.5°/s at 18°.

### Quantitative Characteristics of Fixation Abnormalities

#### Saccadic Intrusions

Square-wave SI and MSO were found in both patients (Figure 2C).

**Figure 4:**
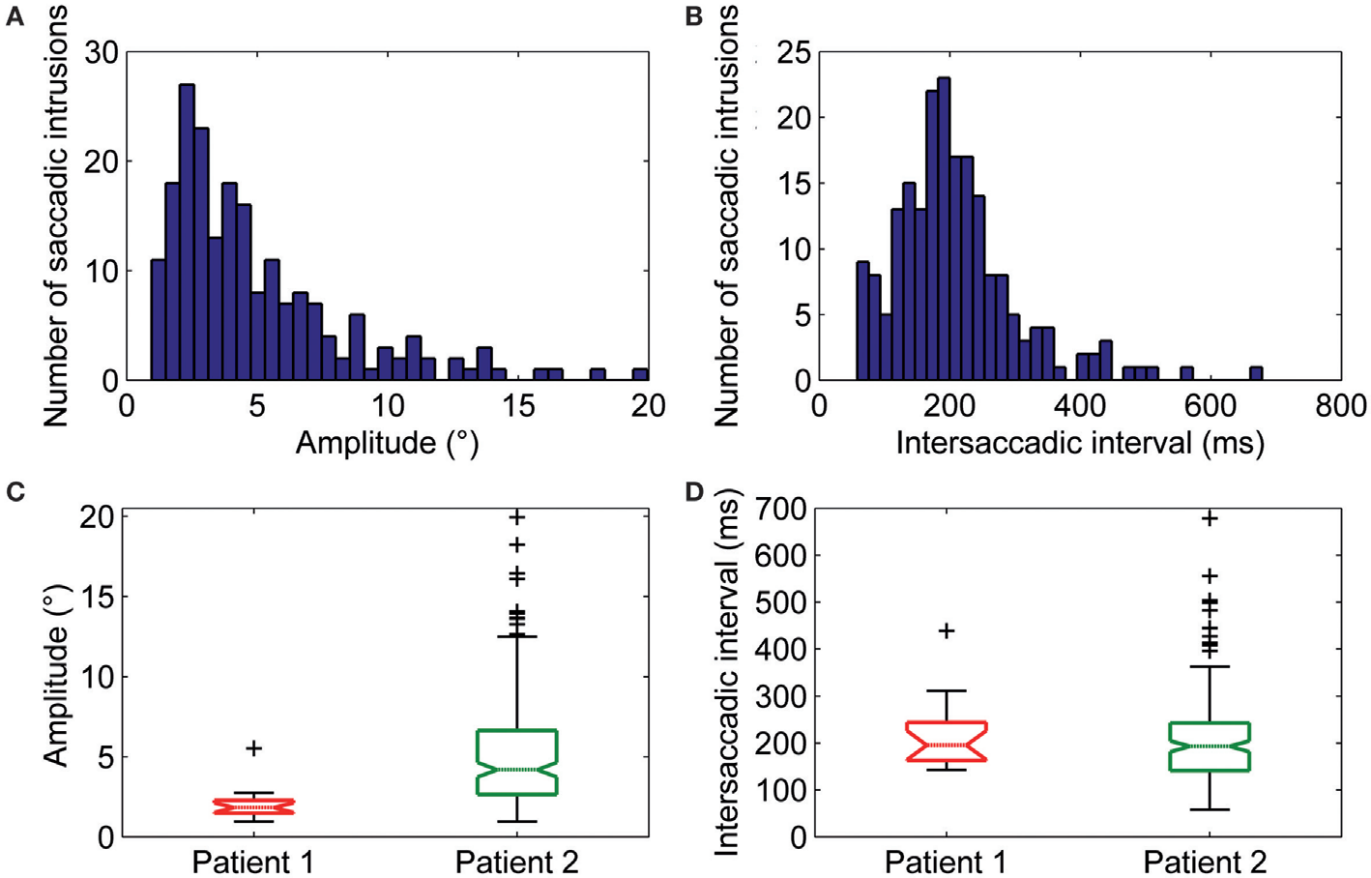
**(A)** Amplitudes and **(B)** intersaccadic intervals distribution of square-wave jerks (SWJs). The *x*-axis in **(A,B)** represents bins of amplitudes and intersaccadic interval, respectively; the *y*-axis represents the number of the SWJs for the given bin. The box and whisker plots show summaries of the amplitudes **(C)** and intersaccadic intervals **(D)** of all SWJs in Patient 1 and Patient 2. The central horizontal bar in each box represents the median, lower, and upper borders are the lower and upper quartile values, whiskers indicate the 95% confidence interval around the median, and outliers (+) are shown individually.

Square-wave jerk showed significantly larger amplitudes (*P* = 0.002) and abnormally higher frequencies than in healthy subjects. The average amplitude of SWJ was 3.6 ± 2.2° (normal amplitude in healthy subjects: 0.7 ± 0.5°) (19). Patient 2 showed SWJ, mainly macrosquare-wave jerks, with higher amplitude. The average intersaccadic interval was 212 ± 86 ms (normal interval in healthy subjects: 255 ± 147 ms) (19). Figures 4A–D show the distribution and a summary of amplitudes and intersaccadic intervals of SWJ in each patient. The frequency of SWJ was 5 intrusions/min in Patient 1 and 48 intrusions/min in Patient 2 (normal rate in healthy subjects: 12 ± 12 intrusions/min) (19). We found BSWSI only in Patient 2, which had larger amplitudes, greater intersaccadic intervals, and higher frequencies than healthy subjects. The average amplitude of BSWSI was (i) 7.6 ± 4.4°, (ii) 14.4 ± 7.1°, and (iii) 6.2 ± 3.3° for away, overshoot, and return saccades, respectively [normal amplitudes in healthy subjects: (i) 0.5 ± 0.2°, (ii) 1.1 ± 0.6°, and (iii) 0.7 ± 0.4°] (19). The average of first and second intersaccadic intervals was (i) 161 ± 74 ms and (ii) 160 ± 65 ms, respectively [normal intervals in healthy subjects: (i) 52 ± 24 ms and (ii) 124 ± 67 ms] (19). Finally, the frequency of BSWSI was 5 intrusions/min (normal rate in healthy subjects: 1 ± 3 intrusions/min) (19).

Macrosaccadic oscillations were especially prominent in Patient 2. Their amplitude ranged between 2.5° and 14.5° with an average value of 7.3 ± 3.3° and their mean intersaccadic interval was 188 ± 46 ms. The frequency of oscillation was 1.9 Hz.

#### Nystagmus

Spontaneous jerk nystagmus, gaze-evoked, and rebound nystamus were present in both patients (Figures 2D–G).

**Figure 5:**
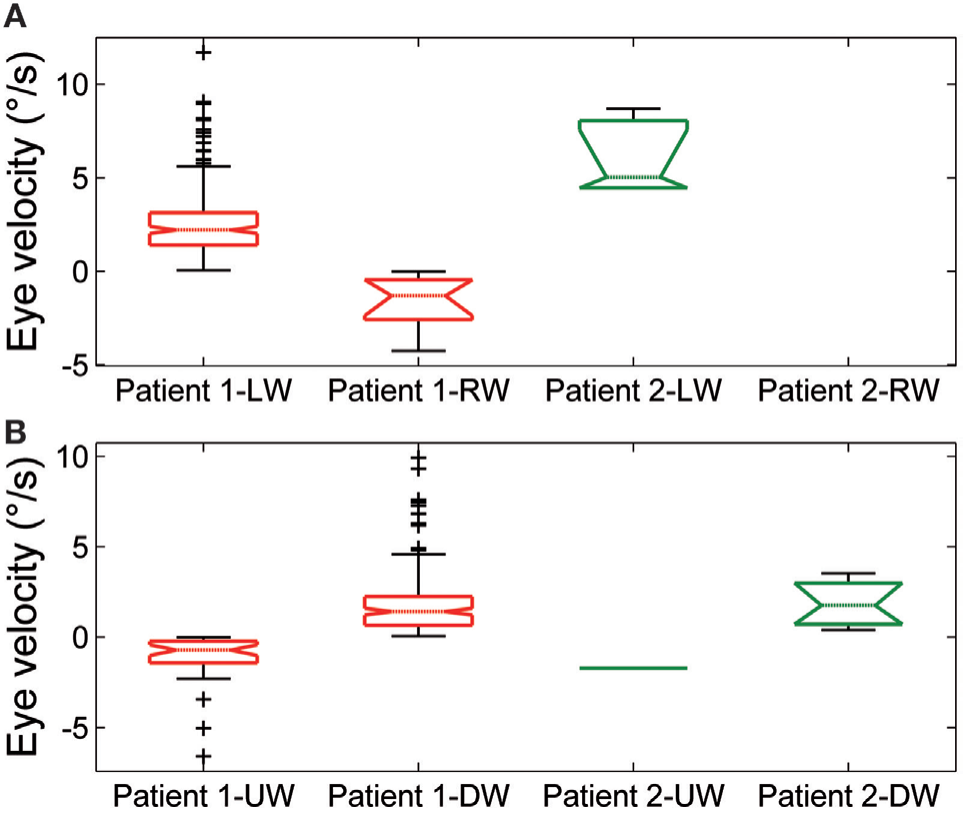
Summary of slow phase eye velocity of **(A)** horizontal and **(B)** vertical spontaneous jerk nystagmus. The box and whisker plots show the slow phase eye velocity of spontaneous jerk nystagmus in Patient 1 and Patient 2. Rightward (RW) and leftward (LW) horizontal nystagmus and upward (UW) and downward (DW) vertical nystagmus are shown separately for each patient. Box plots as in Figure 3.

Spontaneous jerk nystagmus (Figures 2D,E) was mainly horizontal. Sometimes it presented a vertical component that, when it was in phase with the horizontal component, lead to an oblique nystagmus. Horizontal spontaneous nystagmus was left-beating in both patients, with a relatively constant slow phase velocity; the frequency was 1.0 Hz. Figure 5A summarizes the horizontal slow phase velocities. The amplitude was 1.8 ± 1.1° in Patient 1 and 3.1 ± 0.7° in Patient 2. Vertical spontaneous nystagmus was down-beating in both patients. Its slow phase eye velocity is summarized in Figure 5B. The amplitude was 1.1 ± 0.8° in Patient 1 and 1.0 ± 0.6° in Patient 2; the frequency was 0.8 Hz. The direction of the slow phase did not show a substantial change in any patient, although a little modulation in slow phase velocity appeared in Patient 1. Modulation showed a period of 40 s with an amplitude of 0.7° in horizontal nystagmus, and a period of 40 s with an amplitude of 0.4° in vertical nystagmus.

Gaze-evoked nystagmus was present in eccentric gaze positions of 10° and 18° (Figure 2F). The average velocity of the slow phases drift was 4.1 ± 1.9°/s with an amplitude of 2.2 ± 1.2°, and there was a gradual decay in velocity and amplitude during the 30 s of recording. Frequency of nystagmus beats was 1.4 Hz. After the eye returned to central position, a rebound nystagmus occurred in both siblings (Figure 2G). The rebound nystagmus showed a slow phase with an amplitude ranging 0.6°–4.2° and a velocity ranging 0.8°–8.2°/s. Frequency of the rebound nystagmus was 1.7 Hz. Both velocity and amplitude of slow phase drift showed a substantial reduction over 12 s.

## Discussion

This study shows fast, hypermetric saccades sometimes preceded by slow eye movements, SI, MSO, and different types of nystagmus (spontaneous jerk type, gaze-evoked, and rebound nystagmus) in two siblings with ATLD.

Overall, these results confirm that oculomotor alterations are common to ATLD and AT including slow eye movements (drifts), especially following saccades in AT, SI, and different types of cerebellar nystagmus (21–23). Studies on AT patients have demonstrated a severe impairment of gaze fixation stability and VOR, providing elements in favor of a prominent role of Purkinje cells (PC) degeneration in the disinhibition of deep cerebellar nuclei, including the caudal fastigial oculomotor region (FOR), and vestibular nuclei (VN) (21). The loss of GABAergic inhibition on VN can cause nystagmus, including periodic alternating nystagmus (PAN), while disinhibition of FOR can result in instability of the feedback loop projecting to the saccadic burst neurons, leading to SI and oscillations, but also affect the saccade generating mechanisms (21). These abnormalities may explain the postural instability and the impaired gaze fixation due to nystagmus and ocular oscillations not only in AT but also in ATLD patients.

Slow drifts before or after saccades are characteristically seen in AT. Differently to AT patients who may exhibit pre- and post-saccadic drifts, our ATLD patients showed only pre-saccadic drifts. However, the dynamics of the drifts were comparable in AT and ATLD, both showing long duration and relatively linear velocity profiles (22). Slow drifts following saccades have been well characterized in AT, yet different but inconclusive explanations have been proposed to clarify the origin of these movements. They have been attributed to vestibular slow phases; abnormal VOR cancelation (head-free conditions); anticipatory pursuit; post-saccadic drift due to uncorrected pulse-step mismatch by a damaged flocculus, leaky neural integrator causing centripetal drifts, or aberrant suppression of burst cells by omipause neuron triggering slow saccades (22).

Eye movement defects, instead, have never been quantified in ATLD, although clinical qualitative inspections have documented ocular apraxia, SI, spontaneous nystagmus, gaze-evoked nystagmus, and down-beat nystagmus (7). Actually, the significant number of larger and faster LW saccades represents a distinctive oculomotor feature in our patients with ATLD (23, 24) suggesting a major damage of the cerebellar neural network controlling saccade amplitude in these subjects.

**Figure 6:**
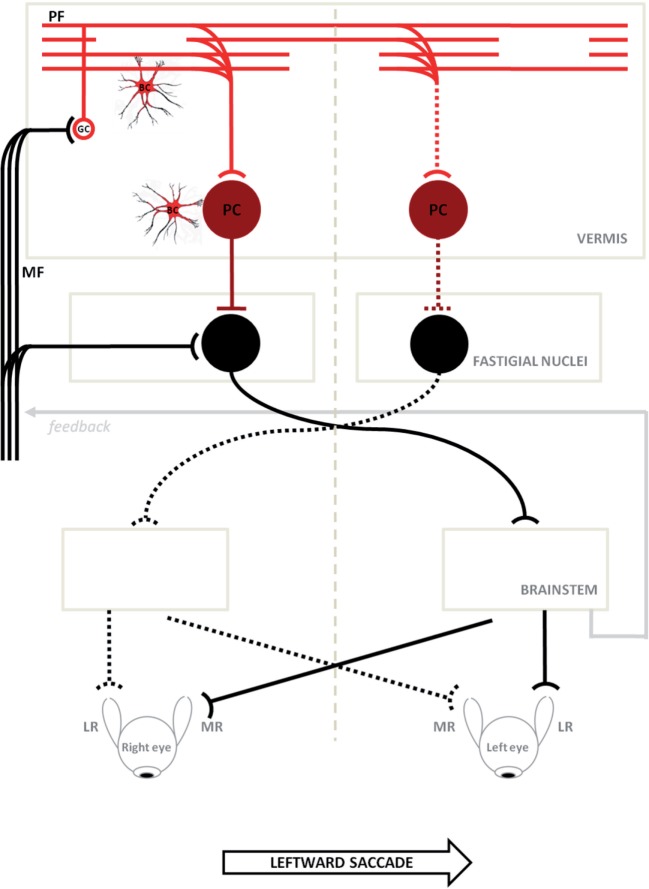
Schematic circuitry for generating horizontal saccades (major active pathways are shown) is hypothesized to explain the disorder of our patients. Projections with curved endings are excitatory, while projections with flat endings are inhibitory. Bilateral input to the cerebellar cortical vermis goes from granule cells (GCs) to the caudal fastigial neurons through parallel fibers (PFs) and Purkinje cells (PC). The PC inhibit the fastigial neurons, canceling out the excitatory drive from the mossy fibers. Just before a horizontal saccade is triggered, neurons in the caudal fastigial nucleus (cFN) of the contralateral side with respect to the direction of the movement, discharge a burst of activity driving the excitatory burst neurons in the brainstem, which in turn drive the eyes in a contraversive saccade (continuous line circuit); later, just before a saccade is stopped, neurons in the opposite cFN burst, in order to decelerate and stop eyes exactly on target (broken line circuit). In ataxia-telangiectasia-like disorder, we consider a double pathogenetic effect that may explain its peculiar saccadic behavior: (1) a developmental anomaly principally affecting Bergmann cells associated with a reduction of the expansion of the GC–PF (light red circuit); (2) a neurodegenerative processes further affecting this circuit including PC synapses (dark red circuit). Slowing of parallel fibers conduction may delay the caudal fastigial neurons burst that blocks saccades, making them hypermetric. The damage of the GC–PF–PC synapses also reduces the inhibitory inputs to both caudal fastigial neurons causing abnormally fast contralateral saccades. The model is adapted from Optican and Quaia (31). ML, media recti; LR, lateral recti.

The neural substrate and mechanisms of saccadic motor control have been extensively clarified in recent years (25–27). It has been shown that the superior colliculus (SC), cerebellum, and brainstem participate in a network controlling saccade amplitude and accuracy. The displacement command for a saccade in a specific direction comes from the SC and goes to the pontine reticular formation where lies part of the cellular network responsible for generating the saccadic command. This network receives signals from dorsal cerebellar vermis lobule VII through the caudal fastigial nucleus (cFN). Through GC collaterals and PFs, signals for the control of saccade accuracy contact PC at the GC–PF–PC synapses (28), before reaching the cFN of the contralateral side (29, 30). Just before a horizontal saccade is triggered (14), neurons in the cFN of the contralateral side, with respect to the direction of the movement, discharge a burst of activity. Later, just before a saccade ends, neurons in the opposite cFN burst, in order to decelerate and stop the eyes exactly on target (31) (Figure 6). Support for this mechanism has been shown in monkeys with lesions of cFN that exhibited saccades overshooting the target ipsilaterally to the lesion (32).

We found that LW saccades of our ATLD patients were significantly hypermetric, while RW ones were hypometric at 18° and slightly but significantly hypermetric at 10° and saccades exhibited lower accuracy in both directions. They were faster to the left and their peak velocity was frequently above the upper limit of the interval of confidence of the main sequence for normal saccades.

It has been assumed from empirical models and experimental data (33, 34) that the slowing of PF conduction occurring in some degenerative cerebellar diseases may delay the cFN burst that chokes off saccades, making them hypermetric. Furthermore, damage to the GC–PF–PC synapses, possibly prevalent in the right cerebellar vermis (see Figure 1), would also reduce the inhibitory inputs to the right cFN causing abnormally fast LW saccades (33, 34), such as those observed in our patients (particularly in Patient 2).

The recent advances in the characterization of the molecular basis and pathologic changes underlying *MRE11* mutations (2) allow us to refine this model. Mre11 together with Rad50, Nbs1, and ATM kinase are key components of the signaling pathway participating in cellular response to DNA damage (35). Mutations in these three genes cause, respectively, ATLD, AT, and NBS, which share some common phenotypic features but also show some differences in clinical presentation and evolution, suggesting a diverse pathogenetic role of the three mutations. Unlike in AT and NBS, predisposition to cancer is uncommon in ATLD, while cerebellar involvement is atypical in NBS, which is mainly characterized by developmental anomalies. However, death from malignancy (8), and severe dysmorphisms (11) have been reported in few ATLD patients, widening the spectrum of possible implications of *MRE11* mutations in neurodegenerative as well as developmental changes. Postmortem studies of patients with ATLD (9) have demonstrated severe cerebellar atrophy, particularly in the vermis and medial part of the hemispheres, while other parts of the brain appeared normal. The number of GC and PF, Bergmann glial cells (BGC) and PC were dramatically reduced (reactive gliosis was very scarce), conversely neurons in the cerebellar cortex of the floccular and nodular lobe, deep cerebellar nuclei, brainstem, and olivary nuclei remained well represented as well as the cerebellar white matter. Moreover, intense immunoreactivity for DNA oxidative stress was evident in granule and BGC (9), suggesting an active neurodegenerative process. This pathologic substrate is slightly different with respect to that reported in AT consisting of a sharper loss of PC with abnormal residual PC often bigger and ectopic (36), but more preserved GC and volumetric density of PF varicosities, and limited qualitative and quantitative abnormalities in the granular layer (37). Changes in deep cerebellar nuclei, olivary nuclei, and cerebellar white matter have also been found in AT brains (38, 39).

According with this pathological substrate, our hypothesis supports the prevalent damage of the GC–PF–PC synapses and provides a possible explanation of the abnormal saccadic behavior observed in ATLD patients.

Here, the available MRI scan (Figure 1) shows extended atrophy of the hemispheres and of the cerebellar vermis, yet the technique does not allow elucidating further details such as, for instance, on the integrity of the cFN.

Hence, based on the results of previous postmortem studies (9) we will assume that the fastigial nuclei in our patients are spared. In this scenario, the findings of hypermetric LW saccades could be explained by hypothesizing that the reduced vermial inhibition is asymmetric, with the right fastigial nuclei being less inhibited by the greater extent of the damage on the right cerebellar vermis. The right fastigial nuclei would then be hyper active and this would cause the saccades to be programmed as excessively large toward the left (27, 34) as the pre-saccadic activity from the right cFN to the left EBN would be abnormally high. The imbalance in cFN activity, or the slowing of PF conduction could also delay, or impair, the intervention of the ipsilateral cFN that would normally stop the saccade on target. During RW saccades, instead, the hyperactive right cFN, lacking proper cerebellar inhibition, would turn off the saccades too soon making them hypometric, although this reasoning would not explain why smaller RW saccades were not following a similar behavior.

Alternatively, a further hypothesis could be related to the role of the cerebellar vermis hypothesized in a recent work by Optican and Pretegiani (40), which considered that this structure, acting as a spatial integrator, determines when to stop a saccade by releasing the ipsiversive cFN from inhibition. The pause in the activity of the contraversive (right) vermis, identifying the target of the saccadic movement, should then spread to the ipsiversive (left) vermis to stop the saccade, and this mechanism, could have been damaged in our patients with ATLD by slowing the PF transmission within the cerebellar vermis, thus delaying the left cFN activation and making saccades hypermetric (33).

Clearly, if the hypothesis of spared FN was disproven, then the classical explanation of a more pronounced dysfunction of the left cFN, which would then cause hypermetric LW saccades, should be considered instead (32).

Moreover, the relative structural preservation of the nodulus-uvula region may justify the absence of PAN in these patients, wherein PAN has been reported in almost all patients with AT (20). The evidence of SWJ with higher amplitude, i.e., more than 7°, is also peculiar in our patients with respect to similar changes reported in AT and other cerebellar diseases. SWJ may seldom be correlated with quick phases of nystagmus in the orthogonal direction (i.e., down-beat nystagmus can have horizontal SWL), but we did not find any correlation.

The proposed hypothesis, based on the circuitry depicted in Figure 6, incorporates another cellular element, namely the BGC, which may be damaged early in ATLD. The BGC have a dual role in normal conditions: they provide a scaffold for the migration/differentiation of GCs in the developing cerebellum, and regulate PC functions in adulthood (41, 42). In this respect, the conditional inactivation of Mre11 determines early cerebellar atrophy and embryonic lethality in murine models (43). We suppose that an early dysfunction of BGC could be responsible for the abnormal development of GC and PF with primitive damage to the GC–PF–PC synapses, later the cerebellar damage may slowly progress due to the overlap of neurodegenerative processes leading to the reported changes (44).

In conclusion, we have reported two Italian adult patients with *MRE11* mutation as the only ATLD patients in whom the eye movements have been analyzed. Although slow eye movements accompanying saccades, various kinds of SI and cerebellar nystagmus are similar to those reported in AT patients, they show fast and overshooting LW saccades. The usually milder phenotype and slower neurological progression with respect to AT and these gaze detectable features may help to address the correct diagnosis in patients with familial neurodegenerative ataxias. A dual pathogenetic mechanism, which incorporates neurodevelopmental and neurodegenerative changes, could determine the phenotype observed in this disease.

## Ethics Statement

All procedures performed in studies involving human participants were in accordance with the ethical standards of Local Ethic Committee: Comitato Etico Locale Azienda Ospedaliera Universitaria Senese and with the 1964 Helsinki declaration and its later amendments or comparable ethical standards. The protocol was approved by the local Ethical Committee (EVAlab protocol CEL no. 48/2010). Patients gave their written consent.

## Author Contributions

PF and AR participated in the design of the work. AR and FR were responsible for patient’s clinical data reporting. PF and FR participated in the acquisition of data. PF, FR, and SR participated in the analysis and interpretation of data. PF drafted the manuscript. PF, EP, and AR participated in editing the manuscript. PF, SR, and AR contributed to the manuscript preparation. AR, SR, and AF participated in the revising the manuscript critically. All authors read and approved the manuscript.

## Conflict of Interest Statement

The authors declare that the research was conducted in the absence of any commercial or financial relationships that could be construed as a potential conflict of interest.
